# Catalyzing sustainable development goals through the water-energy-food nexus

**DOI:** 10.1016/j.isci.2025.111902

**Published:** 2025-01-27

**Authors:** Luxon Nhamo, Sylvester Mpandeli, Stanley Liphadzi, Tafadzwanashe Mabhaudhi

**Affiliations:** 1 Water Research Commission of South Africa, Lynwood Manor, Pretoria 0081, South Africa; 2 Faculty of Science, Engineering and Agriculture, University of Venda, Thohoyandou 0950, South Africa; 3 Centre on Climate Change and Planetary Health, London School of Hygiene and Tropical Medicine (LSHTM), London, UK; 4 Centre for Transformative Agricultural and Food Systems (CTAFS), School of Agricultural, Earth and Environmental Sciences, University of KwaZulu-Natal, Pietermaritzburg 3209, South Africa; 5 Department of Environmental, Water and Earth Sciences, Tshwane University of Technology (TUT), Pretoria West 0029, Pretoria, South Africa; 6 United Nations University Institute for Water, Environment and Health, Richmond Hill, ON, Canada

**Keywords:** Environmental science, Global change, Environmental policy, Natural resources

## Abstract

Water, energy, and food (WEF) are central to sustainable development as they are vital for socio-ecological and socio-economic sustainability and human and environmental wellbeing. How the three are used and managed is central to either the aggravation of climate change or the enhancement of resilience and adaptation strategies. This mixed transdisciplinary study developed a WEF nexus-based framework to guide strategic policy decisions to catalyze progress toward achieving sustainable development goals. The aim is to guide the cross-sectoral management of resources for sustainable development under climate change, increasing demand, depletion, degradation, and uncertainty. Past and present data on resource management was assessed to comprehend future availability toward achieving sustainable development outcomes for people and the planet. The fundamentals of holistic WEF resources management were assessed, highlighting the significance of transformative, cross-sectoral, and circular approaches in enhancing resource use efficiency and sustainability. This is critical for informing sustainable natural resources management decisions.

## Graphical abstract

**Figure:**
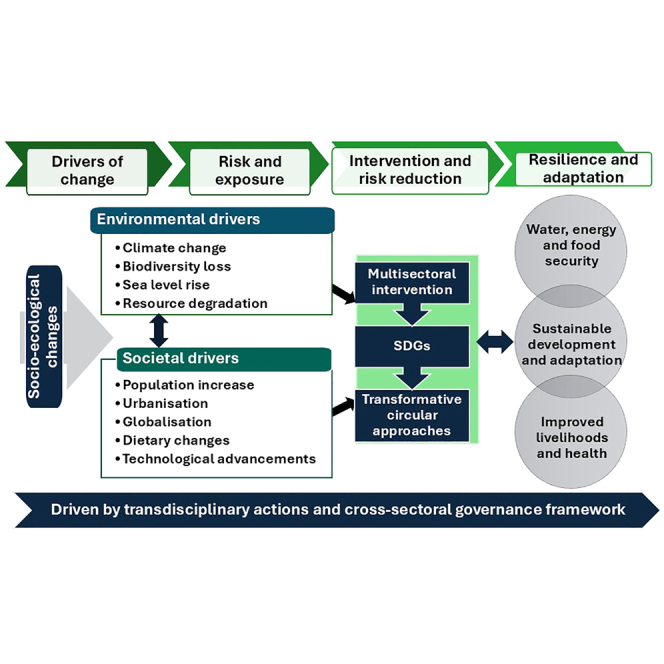

## Introduction

As the second half of the 2030 Global Agenda on sustainable development goals (SDGs) is already underway, it is important to note that developments in the first half indicate a clear and apparent disconnection between global aspirations and reality as there was no meaningful progress in meeting the set targets.^1^^,^^2^ By the halfway mark, only 15% of the SDG targets were on track, and the rest showed stagnation or even reverse mode, an indicator of a stalemate among policy-makers amid multiple crises.^3^ The main challenges hindering SDGs’ progress include (1) the failure by humankind to accept change and accelerate the transition from the norm as they continue in the business-*as*-usual mode, (2) territorial or geopolitical protection in the exploitation of the current unsustainable resources for fear of the unknown, (3) protection of national economies and interests, (4) the increasing frequency of disasters, including the emergence of novel infectious diseases at the global level, and (5) lack of adequate funding to support the transition.^4^^,^^5^^,^^6^^,^^7^ Humankind remains with six years to achieve their SDG targets, with many of the least developed countries (LDC) expected to miss them. This is a concerning trend as poverty, inequality, climate change, and hunger are worsening, and solutions are urgently needed.

As climate extremes continue to increase in intensity and frequency, there is a need to enhance resilience and adaptation and preserve the Earth and its key resources. There is, therefore, a clear consensus to accelerate efforts aimed at making the transformative developmental agenda a reality.^8^^,^^9^ A combination of increasing population, urbanisation, the 4^th^ Industrial Revolution (4IR), and changing consumption patterns further compound the existing challenges of resource insecurity, exerting pressure on an already depleted resource base.^10^^,^^11^ As most SDG targets are already off the mark, there is an urgent need to identify alternative pathways to accelerate our efforts and achieve the desired outcomes within the set time frame; otherwise, the consequences could be dire.^1^^,^^2^

The deteriorating insecurity of water, energy, and food (WEF) resources, together with the slowness of humankind to take decisive measures, are derailing the ambition to achieve the 2030 Global Agenda.^2^ Furthermore, the SDGs need more certainty from sector-based initiatives and linear approaches, yet the targets are interconnected.^12^^,^^13^ The interconnectedness of the SDGs signifies that failure to achieve specific goals will cascade down, causing the failure of the other goals.^1^^,^^14^ Therefore, the current stagnation in SDGs progress is mainly due to focusing on individual sectors, a system emanating from viewing the world from a linear perspective that believes that a single click on a keypad would get the economy and society back on track.^15^ However, focusing on selected SDGs without considering the interlinkages with the other goals generally results in undesired outcomes that include creating suboptimal efficiencies in those specific sectors at the expense of others.^15^^,^^16^

Therefore, the close interlinkages between the SDGs are based on the interconnectedness of WEF sectors as they are at the heart of sustainable development.^17^^,^^18^ The challenges related to the insecurity of the WEF resources formed the basis for the formulation of the SDGs, and the other 14 SDGs are all linked to the three.^19^ The three resources are both culprits and victims of the current socio-ecological and economic challenges facing humankind, hence the three SDGs pillars that include social, economic, and environmental.^20^ As the demand for WEF resources continues to increase, there is a need for policy to consider the broad relations between the WEF sectors and adopt transformative and circular approaches to holistically manage the three resources and ensure sustainability.^21^ The interlinkages between the WEF sectors and their role in achieving the SDGs are manifest through anthropogenic sources of pollution that contaminate the environment and, in turn, degrade water resources.^22^ At the same time, population growth results in increased demand for non-renewable energy resources, which are the major causes of greenhouse gas (GHG) emissions.^23^ Also, to ensure future food security, there should be enough clean water supply for irrigation and energy resources to draw the water from the source to the irrigated fields.^24^ Furthermore, developing and managing the three resources strongly affect global public health.^15^^,^^25^

The intricate interlinkages between the WEF sectors demand transformative and cross-sectoral interventions in their management to enhance synergies and timely address trade-offs.^21^^,^^26^ This is supported by the existing imbalances between the supply and demand of the three resources. For example, at the global scale, water demand has already doubled the rate of population growth, yet almost 30% of the global population still lacks access to safe water, and by 2030, 40% of the global population will suffer severe water stress.^2^^,^^27^ This imbalance is projected to result in almost 700 million people being displaced by intense water scarcity.^28^ Water demand is expected to increase by nearly 55% by 2050, further exacerbating the insecurity of the already depleted and degraded resource.^29^ Similarly, food-insecure people grew from 785 million in 2015 to 822 in 2018 when food production became more water- and energy-intensive.^30^ Agricultural mechanization, intensification, and the increased use of agrochemicals on expanded irrigated land have increased water and energy use in agriculture.^31^ As a result, over 30% of the global energy and more than 70% of the available freshwater withdrawals are used in the agriculture value chain.^32^^,^^33^ While nearly a billion people do not have access to electricity, global energy demand is projected to increase by 25% by 2040, compounding energy insecurity.^34^^,^^35^ As the agriculture sector is under pressure to meet the growing demand for food from an increasing population, there is also pressure to improve the efficiency of water and energy use in the sector.^36^ A combination of these challenges is risking the universal achievement of the SDGs.^2^

The present outlook of the supply of WEF resources to meet demand from the growing population, and at the same time, maintain a sustainable socio-ecological and economic environment and achieve the SDGs looks gloomy.^2^ Of the 169 targets, only 140 have been assessed halfway through the implementation of the SDGs, of which half of them indicate moderate or severe deviations from the desired trajectory, and 30% have shown little or no progress or even regression below the 2015 baseline.^2^ The lack of progress toward achieving the SDGs is mainly due to a lack of commitment and other barriers that include the slow pace of transition to the circular economy, geopolitical reasons, and disasters, among others.^4^ These drawbacks were compounded by the COVID-19 pandemic, which stalled three decades of progress in reducing extreme poverty as all resources were redirected toward the health sector.^37^ If the trend persists, almost 575 million people will remain trapped in extreme poverty by 2030, and around 84 million children will not be receiving formal education.^2^ Global temperature has already reached 1.1°C above the pre-industrial levels and is likely to surpass the critical 1.5°C tipping point by 2035.^9^ The poorest countries and the most vulnerable communities already bear most of the consequences as they lack the resources to adapt.^2^

Given the importance of WEF resources in achieving sustainability, this study proposes pathways to accelerate progress toward achieving SDGs by 2030 through holistic and integrated management of the interlinked WEF sectors amid climate change, increasing demand, depletion, and degradation. The study highlights the interrelations and interconnectedness of SDGs and the need for an inclusive and integrated governance framework as pathways toward the realization of the SDGs. The aim is to provide a detailed narrative of past and present WEF resource availability and accessibility to understand future availability and develop a framework to guide strategic decisions on integrated and sustainable resource management. The study emphasises the significance of SDG data in providing sufficient information for the sustainable management and monitoring of WEF resources. The initial focus was to understand the fundamentals of WEF resources and what was lacking during the implementation of the SDGs during the first half, information that was used to formulate pathways to guide coherent strategic decisions and cross-sectoral recommendations to accelerate progress toward meeting the SDG targets by 2030.

## Linking WEF nexus and SDGs historical background

The WEF nexus became more prominent since 2011 when it was presented at the World Economic Forum (WEF) by the Stockholm Environment Institute (SEI),^38^ a period that coincided with the formulation of the SDGs just before their introduction in 2015.^39^ This was motivated by global projections indicating that the demand for water, energy, and food would increase significantly in the coming years due to population growth, economic development, international trade, urbanization, depletion of the natural resource base, technological advances, and climate change.^40^^,^^41^ It was projected that the demand for the three resources would outstrip supply if no action is taken.^39^^,^^42^ This was also a period when it was firmly established that the three sectors of water, energy, and agriculture (agriculture being a proxy of food) are intricately interlinked and that they form the basis of sustainable development.^43^^,^^44^ This is because the WEF resources are critical for human wellbeing and environmental health, are vital for people and the planet, and their sustainable management would result in desired outcomes including sustainable development.^45^ Thus, SDGs 2, 6, and 7 which focus on ending hunger, access to water and sanitation, and access to clean energy, respectively, are key to the SDGs, and the other 14 goals are linked to the three.^43^ Any development in any one of the three sectors that does not realize the interlinkages with the other sectors and does not consider the environmental impacts would result in undesired outcomes, maladaptation, or just transfers the challenges to the other two sectors.^21^^,^^40^

**Figure 1:**
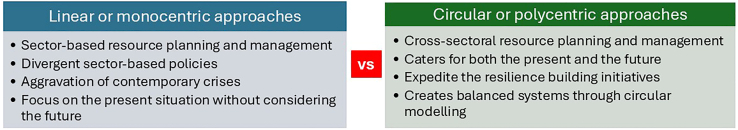
A comparison between linear and circular approaches in managing resources and achieve their sustainability and security

Therefore, the close interlinkages between the WEF sectors prompted the emergence of novel concepts and transformative models, including WEF nexus, scenario planning, just transition, horizon scanning, one health, and sustainable food systems, among others that systematically manage the three resources holistically and capable of informing strategic decisions on avoiding policy spillovers and manage synergies and trade-offs.^46^ This motivated the transition from the current linear approaches that have been creating optimum efficiencies in certain fields at the expense of other equally important sectors^13^ (Figure 1).

The interconnectedness of WEF resources manifests as agriculture accounts for about 70% of total global freshwater withdrawals,^40^ and the sector also uses about 30% of the total energy consumed globally.^47^ Water is equally importance in crop and energy production and transportation in different forms.^48^^,^^49^ On the other hand, energy is essential in the production, transportation, and distribution of food, as well as for the abstraction, storage, and treatment of water.^50^ However, due to the increasing demand for the resource, it is projected that 60% more food will have to be produced by 2050 to meet the food requirements of a growing global population,^51^ energy consumption will increase by over 50% by 2035 ^48^, and freshwater withdrawals for irrigation will increase by 10% globally by 2050.^40^ The recognition of the close interlinkages between the WEF resources and the need for transformative and circular models to guide the simultaneous management of the three resources promoted the WEF nexus to be widely studied in the last decade.

**Figure 2:**
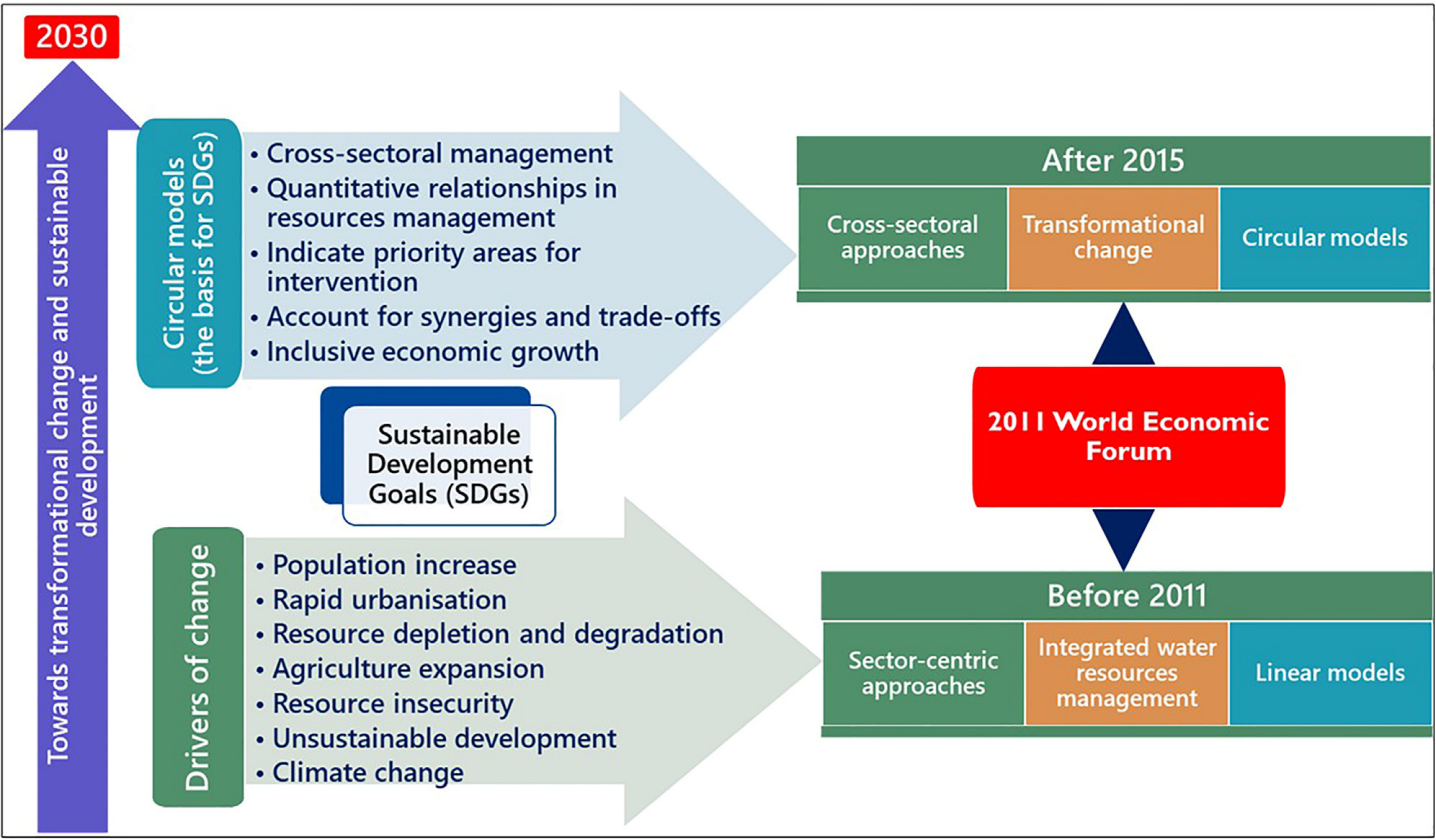
An illustration showing the distinct focus areas of the WEF nexus concept before and after 2015 The illustration also indicates the factors driving the WEF nexus and how it can be used to achieve the SDGs. Source*.*^52^

The WEF nexus concept existed before the enactment of the SDGs in 2015, but there is a clear demarcation of the WEF nexus before and after the enactment of the SDGs in 2015. Before 2015, the concept focused on individual sectors and sector-specific models with no or limited integration^41^^,^^52^^,^^53^^,^^54^ (Figure 2). This viewpoint maintained the linear economy approach (Figure 2), which aggravates contemporary crises by encouraging optimal development of certain sectors at the expense of the other interlinked sectors.^55^^,^^56^ However, the linear viewpoint has since transitioned to a more people, planet, and environment-focused transformative and circular model, the WEF Nexus, which considers intergenerational concerns.^13^ The approach has evolved into an important tool for assessing and catalyzing SDG targets implementation.

## Applied systemic processes and actions

**Figure 3:**
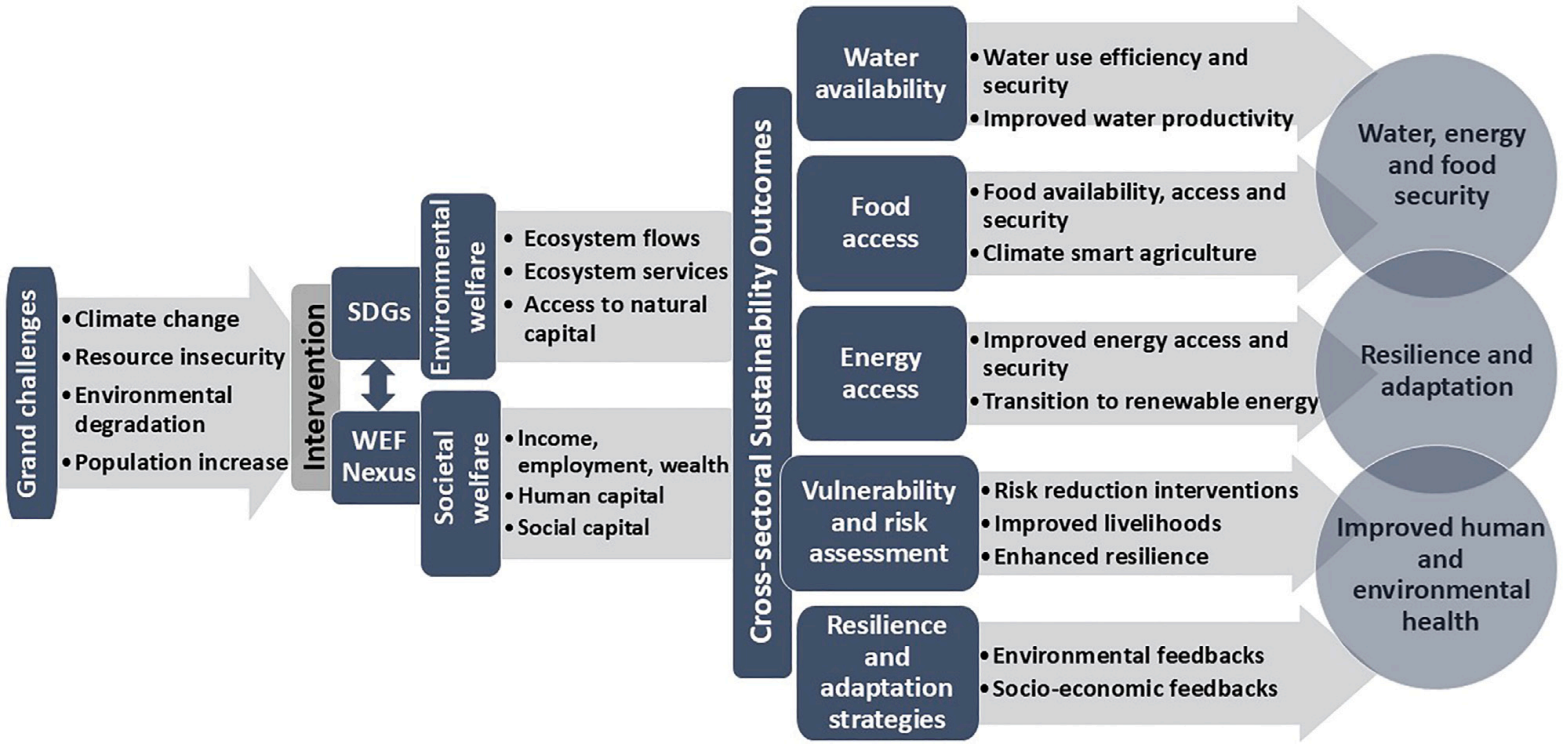
A stepwise methodological framework illustrating the systemic processes and adaptation targets and action fields toward achieving sustainability, including the background of the sustainable development goals, the interventional actions that need to be adopted to address the challenges, the cross-sectoral transformative approaches to be applied, and the envisaged sustainability outcomes derived from integrated and cross-sectoral interventions

This study used a transdisciplinary approach to answer specific research questions, including: (1) how have WEF resources been managed since 2015? (2) What is the progress toward achieving the SDGs? (3) What are the prospects of catalyzing progress toward SDGs in the second half of implementation? and (4) What have been the major challenges derailing the realization of SDG targets? The assessment then resulted in the development of a framework that guides strategic policy decisions that accelerate progress toward the realization of the SDGs. Figure 3 illustrates the methodological framework used to achieve the study’s goals, indicating the main action fields. The approach (Figure 3) facilitated the formulation of a proposed conceptual framework to guide coherent strategic decisions that lead to sustainable development under a myriad of challenges, including degradation, depletion, increasing demand, and climate change. The approach facilitated the integration of inductive and deductive information, enabling theory generation and hypothesis testing required in transformative approaches.

The first part was a review of selected international literature that included policies, frameworks, and strategies, among other publications of significance to the theme of the study. The articles were searched from Google Scholar, Scopus, and Web of Science using search terms like “achieving sustainable development goal”, “progress on sustainable development goals”, “WEF nexus and SDGs”, “challenges to achieving SDGs”, “SDGs governance frameworks”, “progress on ensuring water, energy and food security”, “interlinkages among the SDGs”, and “prospects on realizing the SDG targets”.

**Figure 4:**
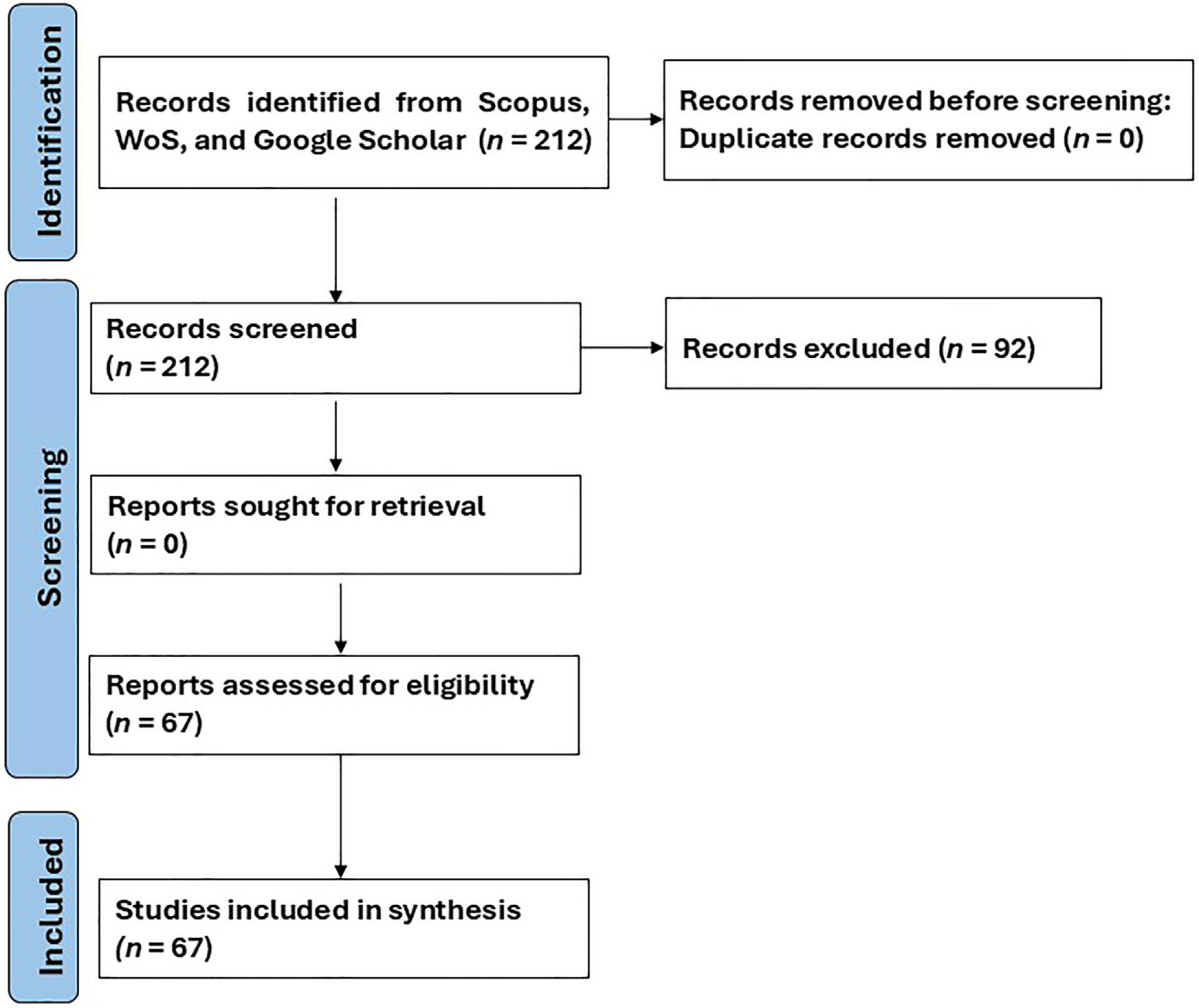
Graphic representation indicating the phases of the literature search, handling, and screening

Both peer-reviewed and gray literature were considered during the search resulting in over two hundred articles that were retrieved. The number was reduced to sixty-seven articles that were finally considered to be relevant to the objectives of the study. Figure 4 is a schematic flowchart indicating the process followed during the literature search and screening, which yielded *n* = 212 articles, and after a thorough screening, *n* = 145 articles were excluded. This resulted in *n* = 67 articles being considered for synthesis.

The second part involved the application of the integrative analytical WEF nexus model^21^ to assess progress made in the first half of the implementation of the SDGs. The progress toward SDGs was achieved by comparing WEF resources security and management between 2015 and 2020, taking the example of South Africa. The integrative analytical model applies the analytical hierarch process (AHP), a multi-criteria decision making (MCDM) process developed by Saaty.^57^ The model integrates WEF resources security-related indicators through the pairwise comparison matrix (PCM) to develop quantitative relational composite indices. The indices are then used to establish the numerical relationships between the WEF resources through a spider graph.

Lastly, an analysis of the information derived from the literature review and the integrative analytical WEF nexus model was integrated to comprehend the stagnation of the SDGs and the hindrances impeding the transition to sustainability. This knowledge facilitated the development of an inclusive, innovative, cross-sectoral, and transformative conceptual framework to drive the SDGs during the second half of their implementation. The essence of the framework highlights the importance of transformative approaches (WEF nexus, circular economy, scenario planning, one health, sustainable food systems, and just transitions) in managing resources holistically and sustainably amid the current cross-cutting grand challenges of climate change, environmental degradation, and population increase. This transdisciplinary approach provides a holistic and cross-sectoral assessment of the challenges at hand, allowing for timely intervention in case of trade-off, facilitating the reduction of uncertainties.

## Developing the conceptual framework

The information derived from the reviewed documents was used to develop a framework providing strategic cross-sectoral policy decisions on managing and developing the interlinked WEF sectors. This is done to simultaneously achieve the desired outcomes across the sectors. The process facilitated the identification of interconnected core themes that concurrently drive SDG implementation. This was informed by the knowledge that the SDGs are interlinked and that failure of one goal has the potential to impact the rest of the goals or focusing on individual goals creates suboptimal efficiencies in those goals at the expense of the others.^6^^,^^17^ The framework also recognizes the transdisciplinary nature of water, energy, and food and that the three sectors are at the heart of all the SDGs.^39^^,^^58^

A key outcome of the framework is an outline for a multisectoral, multicentric, transdisciplinary, transformative, and integrated resource management for inclusive and transformational sustainable development.^13^ The framework is, therefore, a roadmap that leads toward inclusive, sustainable development, and enhancing resource security. It is an integrated guideline based on a transdisciplinary analysis linked to climate change resilience and adaptation.

## Global WEF resources outlook

Providing water, energy, and food to all at all times in a changing environment, increasing demand from a growing population, and the advent of climate change have become a topical subject in global discourses in recent years. However, indications on the ground paint a gloomy picture of meeting the related SDG targets by 2030 as most of the targets are off track.^2^ As already alluded to, achieving the SDGs related to water, energy and food will catalyze the realization of the other 14 SDGs as the other SDGs are all somehow linked to the three.^19^ Current efforts to achieve sustainability by 2030 are at risk due to the gloomy outlook as statistics indicate that the SDGs remain an ambition as many people still lack access to the three resources and in some instances, the numbers are even increasing.^2^ It is the responsibility of everyone to achieve the SDGs as they represent every stage of the value chain. This realization of cross-sectoral management at every stage is key to achieving the circular economy.

Water resources continue degrading and depleting globally, resulting in demand outstripping supply.^59^ More than 1.7 billion people live in river basins where water depletion exceeds natural recharge due to excessive use, and nearly 80% of the global population lives in water-insecure countries.^60^ In addition, over 10% of the world population (close to 800 million) does not have access to basic drinking water, and more than 70% (close to 5.5 billion) do not have access to a safely managed drinking water service.^60^ Nearly 30% of the global population still needs access to safely managed drinking water, an indicator that Goal 6 needs to catch up.^2^ Besides, the Goal does not only focus on providing drinking water, sanitation, and hygiene (WaSH) services, but it also concerns the sustainable management of water resources globally.^58^ The goal stresses enhancing water access, reducing pollution, managing transboundary water resources, improving water use efficiency and productivity, and curtailing unsustainable water withdrawals.^61^ However, unabated pollution continues to degrade the quality of water resources globally, wetlands continue shrinking at an alarming rate of 0.2% annually, and transboundary cooperation remains restricted and, in most cases, is the cause of conflicts.^62^ These anomalies signify a clear disconnection between global aspirations and reality as we pass the halfway mark without meaningful progress in achieving Goal 6.

From the energy perspective, about 660 million people will be without access to electricity, and close to 2 billion people will depend on polluting fuels for household use by 2030.^2^ The 0.6% growth rate in electricity access between 2019 and 2021 lags behind the 0.8% projected in 2015–2019.^63^ About 29% of the global population (2.3 billion people) still rely on inefficient and polluting cooking systems, risking their health and exacerbating climate change challenges. Non-renewable energy sources contribute around 60% of global greenhouse gas emissions.^23^ The slowness in energy transition contributes to ecological collapse as the sector is key in reducing climate change, accounting for about two-thirds of global GHG emissions.^63^ Greenhouse gas emissions from the energy sector have been increasing by about 1% yearly since 2015, risking the global carbon budget to be exhausted by 2030.^35^ To meet the energy targets by 2030, energy intensity needs to improve by about 3.4% per annum, which could prove difficult in LDCs.^2^

Achieving Goal 2, which is on food security, by 2030 requires a profound change in the global food and agriculture system, which is currently off-track^64^ as global food insecurity has worsened since 2015 due to COVID-19, conflict, climate change, growing population, and increasing inequalities.^2^ Nearly 9.2% (almost 735 million people) of the world population faced chronic hunger in 2022, which is 122 million more people than in 2019, and around 29.6% (almost 2.4 billion people) were food insecure in 2022, that is 391 million more than in 2019.^2^ Although there have been improvements in agricultural productivity in the past years, the sector has struggled to meet the increasing global food requirements.^65^ The increasing productivity in agriculture has come at a price of environmental degradation, water pollution, biodiversity loss, the emergence of novel infectious diseases, GHG emissions, and climate, among other stressors.^66^ The close interlinkages between agricultural productivity and the degradation of the socio-ecological systems call for transformative and circular models to guide integrated assessment and identification of intrinsic properties for timely interventions.

## Prospects to enhance the sustainability of WEF resources

As the grand challenges facing humankind today transverse all sectors, there is a need to transition from a linear economy to a circular one that catalyses transformational change toward greater sustainability, resilience, and equity and delivers on human health and wellbeing, and environmental outcomes.^67^^,^^68^ The WEF nexus fits this dimension as it recognizes the interlinkages between socio-economic and ecological systems and how their systemic properties shape their interactions, interdependencies, and interrelationships.^68^ An added advantage of the WEF nexus is that it integrates, simplifies, and facilitates integrated interventions in resource management, reducing risk and vulnerability,^40^ and therefore forming an integral part of the SDGs.^43^ The assumption is that the sustainable management of WEF sectors catalyses sustainable development, thus enhancing all the other SDGs.

**Figure 5:**
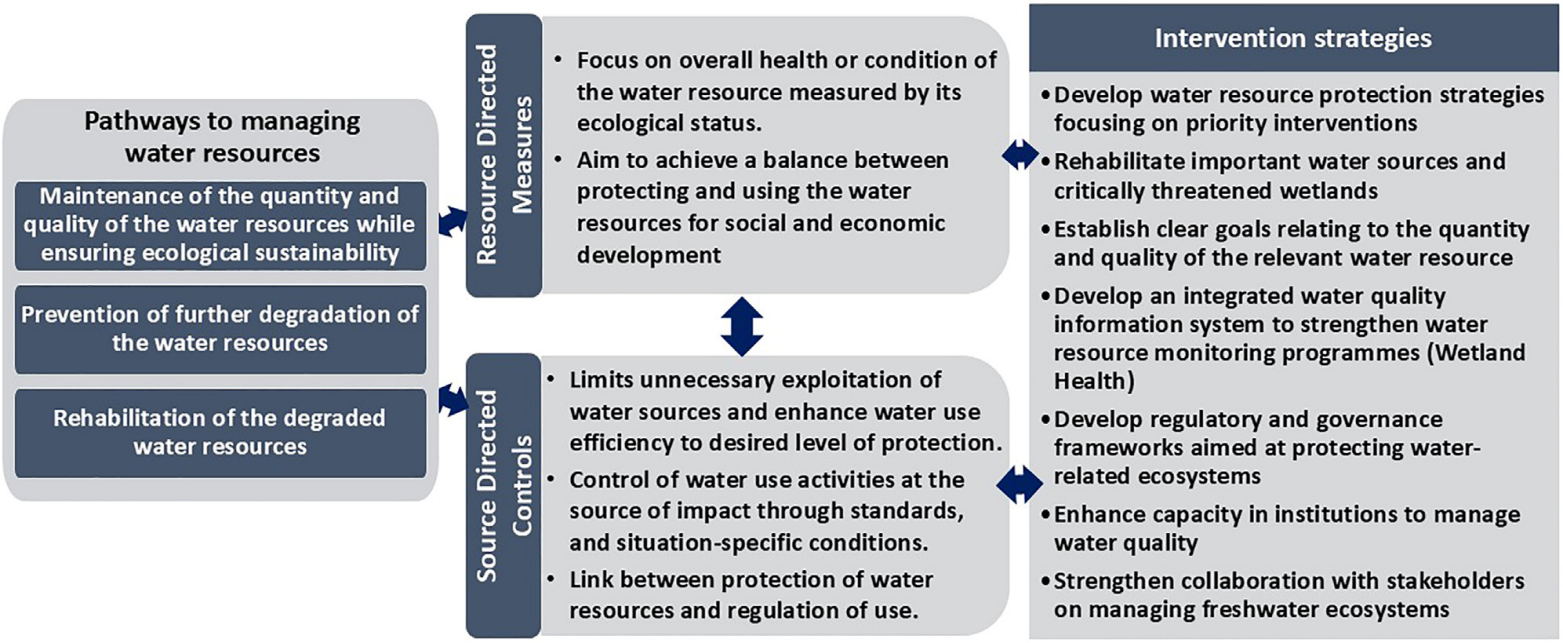
An outline of the pathways to sustainably manage water resources, their drivers and intervention strategies that guide strategic policy decisions toward meeting the SDG 6 as part of a cross-sectoral and transformative approaches

In the water sector, the WEF nexus provides decision-makers with strategic policy decisions on managing water resources, including (1) maintenance of the quality and quantity of water resources while ensuring ecological sustainability, (2) prevention of further degradation of the resources, and (3) rehabilitation of the degraded water resources^69^^,^^70^ (Figure 5). This is critical for the provision of sustainable pathways in managing water resources, including (1) resource-directed measures and (2) source-directed controls (Figure 5). The pathways and sustainable management are driven by interventional strategies that result in coherent policy decisions toward water sustainability and the realization of Goal 6.

From an energy perspective, the WEF nexus guides policy decisions on the formulation of an enabling governance framework that fosters energy access, enhances energy use efficiency, promotes the adoption of climate-smart technologies and system improvements, and directs consumer behavioral change.^71^ This will culminate in a transformative and sustainable energy transition framework that integrates the interlinked actionable interventions including (1) technology and resource efficiency, (2) climate change and a just transition, (3) sustainable energy systems, (4) sustainable land and food systems, and (5) global development, equity, and cooperation. Developing a framework for sustainable energy transition is a complex process that must consider and integrate sustainability’s environmental, technical, social, institutional, and economic dimensions.^72^ Although the energy transition and ambitions face uncertainties in all countries, the challenges are more pronounced in countries whose economies are dependent on fossil fuels.^2^ The sustainable energy transition is driven by robust and WEF nexus-informed interventions.

**Figure 6:**
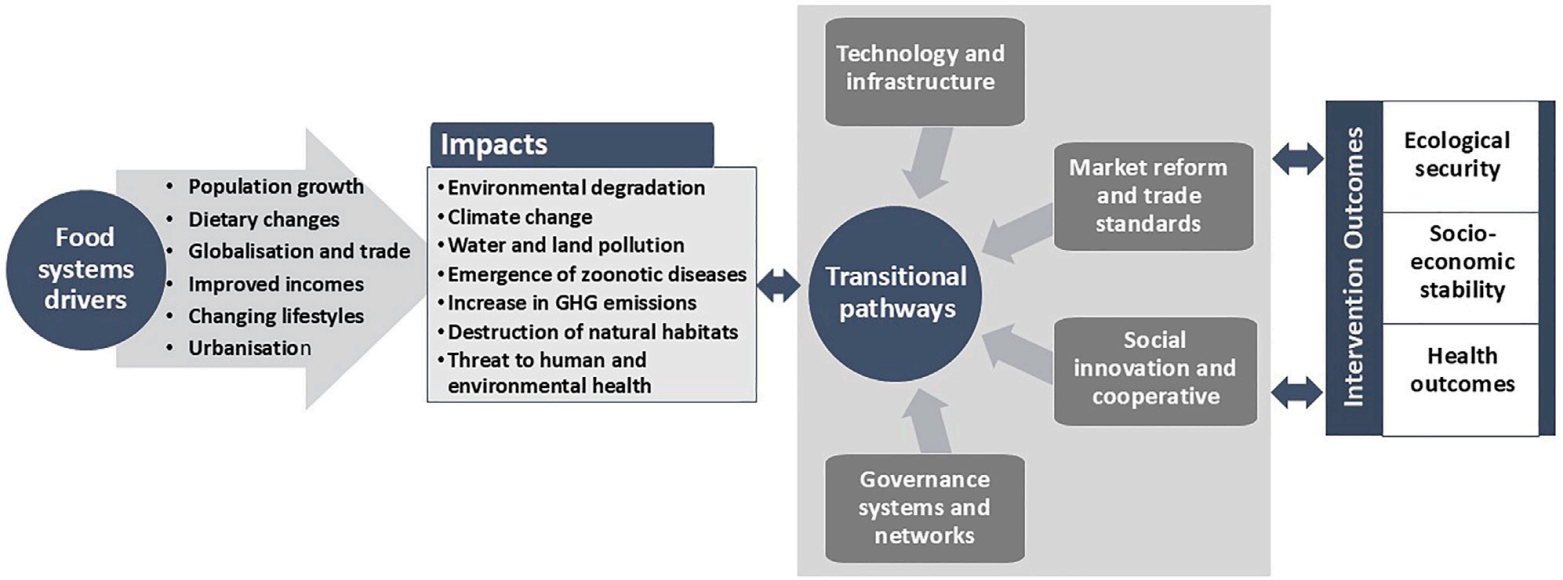
A simplified illustration of the transitional food systems pathways indicating the drivers of change and their impacts, the roadmap to achieve the sustainability in food systems and food security and their benefits that include human and environmental health, socio-economic stability, and ecological security

In the food sector, the WEF nexus is equally crucial for guiding policy decisions on achieving food security and sustainable food systems and the formulation of a sustainable food system framework that entails long-term food and nutritional security for the present and future generations. The benefits are huge although achieving food security is a complex process that requires functional, equitable, and resilient food systems, accompanied by a practical and action-based framework that guides policy and research in building sustainable food systems.^73^ In that regard, the WEF nexus facilitates the iterative multidisciplinary processes that involve all stakeholders and informs transitions that include key components like increased efficiency (sustainable intensification), demand restraint (sustainable diets), and food systems transformations (alternative food systems).^74^^,^^75^ The WEF nexus model guides the transition from an agricultural-centred system, or a water-centric system to one that is polycentric and sustainable and capable of integrating complex processes and transformations (Figure 6). Figure 6 illustrates the pathways and insights that frame the structure, behavior, and performance of the interlinkages between components of food systems.^76^ The framework establishes the broad interlinkages between (1) food system drivers (urbanization, technology development, climate change and economic growth), (2) food system components (production, distribution, packaging, retailing and consumption), and (3) food system outcomes (health, sustainability, resilience, and equity).

Therefore, disregarding the interconnectedness of water, energy, and food sectors when implementing strategies aimed at realizing SDG targets presents the risk of causing undesired outcomes, maladaptation, and stagnated development as what happened during the first half of the SDGs implementation. Little or no progress was achieved as countries pursued specific and preferred goals, a scenario that created optimal efficiencies in a few preferred SDGs at the expense of others.^2^ As already illustrated, the WEF nexus is framed to guide integrated and cross-sectoral implementation of SDGs and to timely indicate priority areas needing intervention.^21^ The significance of the WEF nexus in guiding integrated, simultaneous, and holistic management of interlinked resources triggered its prominence since 2015.^52^ The integrative analytical WEF nexus model developed by Nhamo and colleagues^21^ managed to show WEF resources status and management graphically and numerically at various spatial scales and identified priority areas for intervention.^21^^,^^77^^,^^78^^,^^79^ The WEF nexus analytical model and other recently developed WEF nexus frameworks are being applied to catalyze the SDGs implementation.^21^^,^^80^

## An assessment of SDGs progress: WEF nexus modeling

A synopsis of the status of the security of WEF resources (as informed by resources security indicators) in 2015 and 2020 (Table 1) for South Africa ^81^ is used to demonstrate how the integrative analytical WEF nexus model is used. The AHP was used to establish the PCM, normalization of the indices and provide the numerical relationships between the distinct indicators.^21^ The process, which is applicable at any spatiotemporal scale, was done for 2015 and 2020 and the comparison between the two-period intervals forms the basis to assess SDGs implementation as the WEF indicators are the same as the SDGs indicators. The essence of the approach is that it simplifies the understanding and interpretation of the complex relationships among the WEF sectors, and guides policy decisions on holistic management of interlinked sectors.^13^^,^^21^

**Table 1:** State of the WEF resources indicators for South Africa in 2015 and 2020

Indicator and short name	Indicator status		
	2015	2020	Units
Proportion of available freshwater resources per capita (availability)	821.3	821.4	m^3^
Proportion of crops/energy produced per unit of water used (water productivity)	26.2	26.2	$/m^3^
Proportion of population with access to electricity (accessibility)	85.5	84.4	%
Energy intensity measured in terms of primary energy and GDP (productivity)	8.7	8.7	MJ/GDP
Prevalence of moderate/severe food insecurity in the population (self-sufficiency)	5.7	6.2	%
Proportion of sustainable agricultural production per unit area (cereal productivity)	3.5	5.6	kg/ha

Source: World Bank Indicators (2024).

The PCM and the normalisation of indices were repeated for both 2015 and 2020 to generate the composite indices as shown in Table 2. The indices for each of the indicators represent the quantitative relationships between the WEF sectors indicators, however, they are still difficult to interpret. The quantitative relationships between the indicators are best expressed through a spider graph, which vividly illustrates how resources are related and managed. The WEF nexus integrated index is a weighted average of the composite indices to indicate the level of a country in resource management according to the classification given by Nhamo et al.^21^

**Table 2:** WEF resources security composite indices for South Africa in 2015 and 2020

Indicator	Composite indices	
	2015	2020
Water availability	0.126	0.099
Water productivity	0.128	0.221
Energy accessibility	0.141	0.079
Energy productivity	0.111	0.199
Food self-sufficiency	0.314	0.292
Cereal productivity	0.180	0.111
WEF integrated index	0.203	0.155

**Figure 7:**
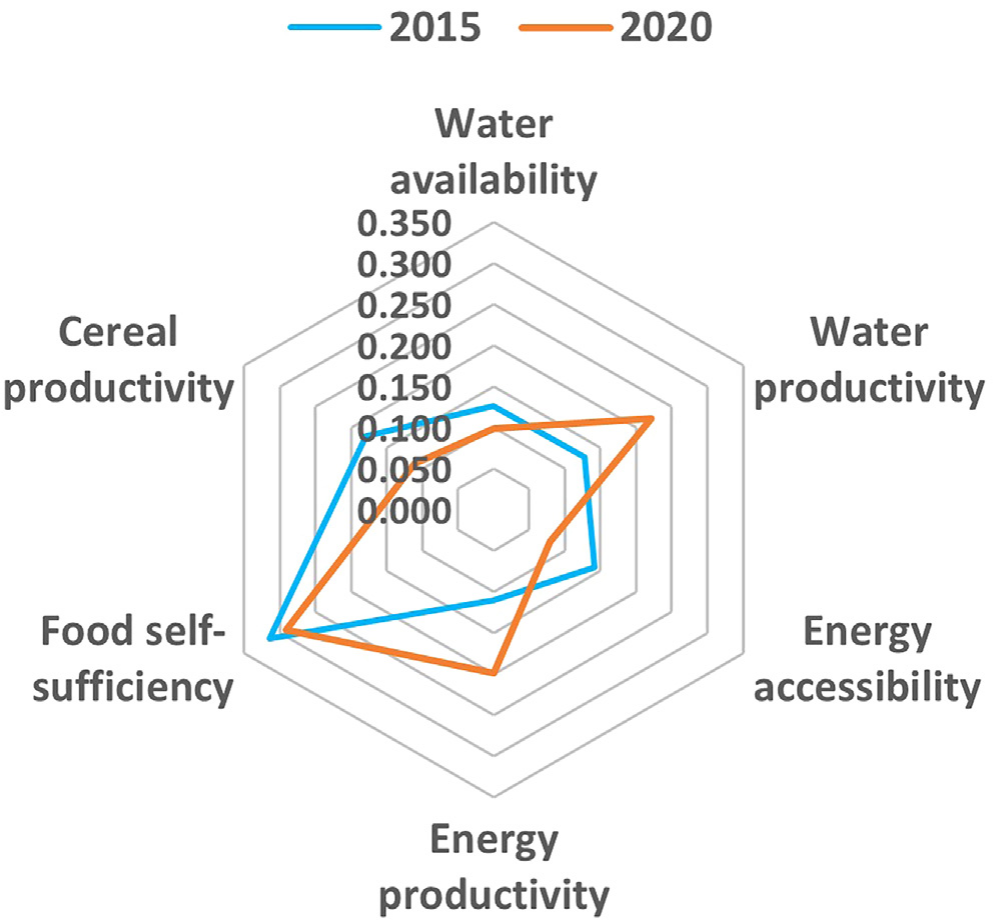
Changes in WEF resources management in 2015 and 2020 in South Africa The comparison facilitates periodic assessment of SDGs progress over time. The deformed centrepieces indicate an imbalanced and unsustainable resource management.

The composite indices (Table 2; Figure 7) derived from the PCM are dimensionless relations ranging between 0 and 1, representing how an indicator is quantitatively related to the other indicators in terms of cross-sectoral and integrated management of interlinked resources. For example, the water availability indicator relates to other indicators by 0.126 in 2015, and this decreased to 0.099 in 2020.^21^^,^^79^ However, an indicator of 1 represents the best possible resource management and 0 represents poor management practices. The relationship between the indicators is best represented and interpreted as a spider graph^21^ (Figure 7).

The spider graph showing the relationships between the WEF indicators for 2015 and 2020 provides the numerical relationships between the WEF resources (Figure 7). The centrepieces, for both years, show deformed relationships, an indication of sector-based and linear management of resources still being pursued. The centrepieces are expected to be circular in shape to indicate balanced and holistic resource management and a clear path toward sustainability. Therefore, the shape of the centrepieces provides an overview of the state of resources management and the models being pursued. The current sector-based and linear approaches in resource management are only exacerbating the contemporary challenges that cut across all sectors and are the main reason for the stagnation of the SDGs. The shape of the centrepieces is also critical for indicating areas needing immediate intervention from a cross-sectoral and holistic perspective. The irregular shapes of the centrepieces for both reference years are an indication that the country is focusing more on selected SDGs targets at the expense of the others.^21^^,^^77^^,^^78^^,^^79^

Therefore, the integrative analytical WEF nexus model is a decision-support tool for assessing the state of resource management at any given time and space. In the case of the SDGs the model can be used to assess SDGs progress for a time interval of five years (2015, 2020, 2025, and 2030), thus offering the potential of monitoring and evaluation of SDG targets. The approach provides pathways to (1) enhance holistic, sustainable, and resource use efficiency of the WEF resources, (2) promote equitable and balanced resource management and distribution, (3) ensure human and environmental health, and (4) support the provision of ecosystem services by guiding the sustainable management of resources. These attributes make the WEF nexus an essential transformative and systems approach to assess the SDGs, promote resource use efficiency, and enhance climate change resilience and adaptation.^13^ Importantly, the approach is applicable at any spatial scale.^21^^,^^77^^,^^78^ However, besides the current evidence of the importance of the WEF nexus, its implementation has been hindered by the lack of a harmonized and holistic governance framework.

## A nexus-based conceptual framework to drive WEF-related SDGs

**Figure 8:**
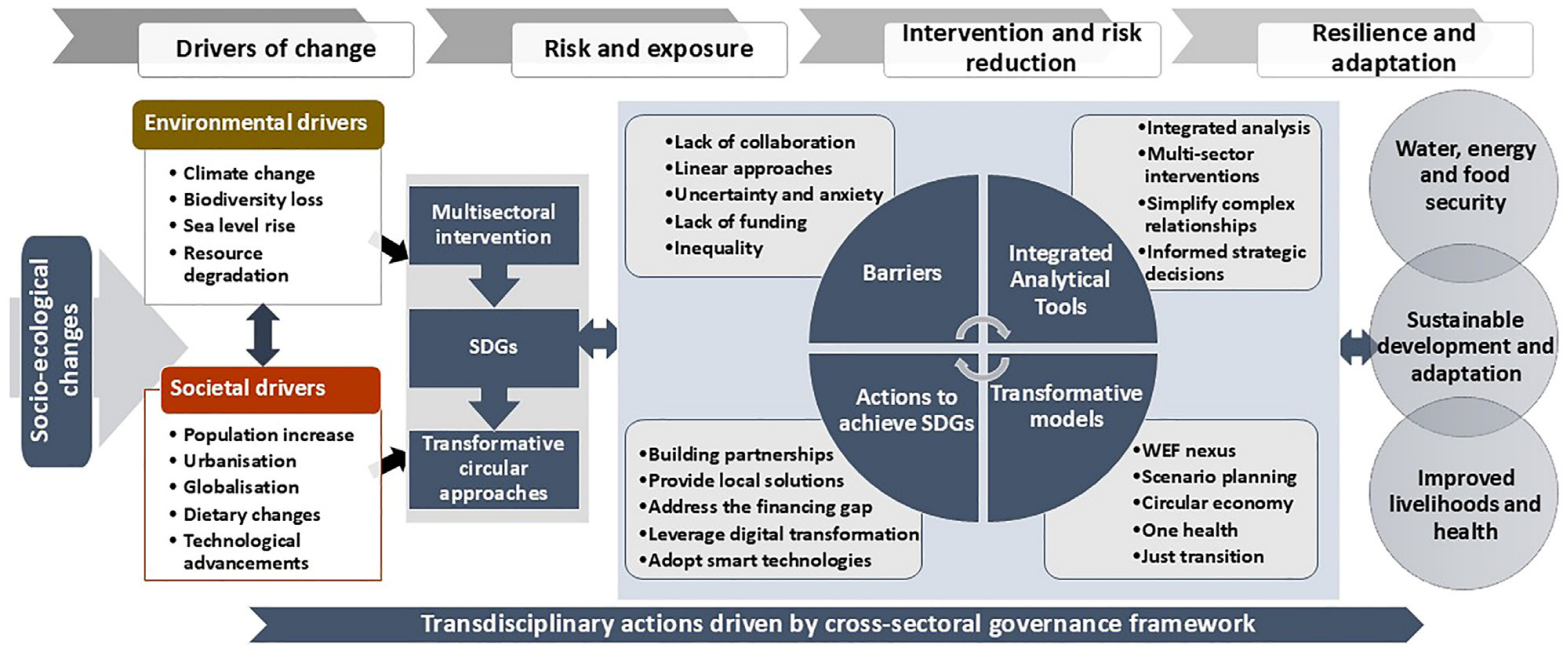
A proposed stepwise nexus-based framework outlining the multisectoral, multicentric, transdisciplinary, transformative, and integrated process for cross-sectoral and integrated management of resources for an inclusive and transformational sustainable development to catalyze progress toward achieving the SDGs

Four main interlinked and transdisciplinary thematic areas drive socio-ecological changes, stimulate the associated vulnerabilities and risks, and provide the pathways that can be adopted to remedy the resulting cross-cutting challenges.^9^ These include (1) the drivers of environmental and societal changes, (2) risk and vulnerability and the capacity to adapt, (3) intervention and risk reduction initiatives, and (4) the resilience and adaptation-building pathways that guide strategic policy decisions (Figure 8). The recognition of the phased key themes and the use of smart technologies and the adoption of transdisciplinary actions enhance the resilience and adaptation initiatives, provide the pathways toward the simultaneous realisation of SDGs. Addressing socio-ecological drivers (Figure 8) requires multi-sectoral interventions through transformative approaches like the WEF nexus that provide pathways toward concurrent and timely responses to the cross-cutting challenges. The integrative analytical WEF nexus tool is an example of a tool that can assess interlinked sectors simultaneously, provide numerical relationships, and inform on priority areas for immediate multidisciplinary interventions to reduce risk and manage synergies and trade-offs. The tool establishes numerical relationships between key indicators and their performance, quantifying the trend for monitoring and evaluation purposes.^21^

The integrative analytical WEF nexus tool guides the actions needed to achieve the SDGs and overcome the pertinent barriers that may impede the realization of the SDGs.^79^^,^^82^ Transformative transitions encounter multiple challenges (barriers) that are addressed by engaging all stakeholders and through the use of integrated analytical tools that drive transformation. The adoption and application of the WEF nexus integrative tool address the challenges and ensure the security of WEF resources, improve human and environmental health and culminate in the realization of the SDGs.^21^ These transdisciplinary actions are driven by a cross-sectoral and multidisciplinary governance framework.

The transdisciplinary themes and actions provide simultaneous responses to the contemporary challenges that cut across all sectors toward the realization of the SDGs. The integration of these key factors informed the development of a WEF nexus-based framework (Figure 8), which responds to each of the thematic areas and guides strategic decisions on sustainable development from a WEF nexus viewpoint. This is particularly important as the transdisciplinary themes require transformative and circular approaches to sustainably intervene and enhance resilience and adaptation.^83^ This formed the initial phase of the transitional pathway toward achieving SDGs. The application of transformative approaches during multisectoral interventions is a transdisciplinary process driven by a cross-sectoral governance structure that recognizes the intricate interconnectedness and interlinkages of sectors.^84^^,^^85^

Systemic, cross-sectoral, and transdisciplinary interventions in resource management and development result in simultaneous benefits and desired outcomes, including resource security, economic development, enhanced human, and environmental health, improved livelihoods, and, ultimately, the realization of the SDG targets.^79^^,^^85^ As already alluded to, the WEF nexus is one such transformative and cross-sectoral approach to catalyze progress toward the realization of SDGs and guides the transition from the current linear model to a circular economy.^86^

## Recommendations

Achieving the SDGs is dependent on how society uses natural resources. However, to monitor the sustainable use of resources, it is imperative to constantly monitor the use, management, availability, and accessibility of the resources with support from national and regional funding agencies. The linear model in resource management is unsustainable, and if it continues unabated, society will run out of resources needed for survival.^2^ An important factor to consider regarding accelerating progress toward achieving the SDGs is recognising water, energy, and food as the basis of SDGs. The three have dedicated goals and the rest of the SDGs are all connected to the three resources. However, present data on the progress of SDGs and the current application methods still lean toward linear approaches and sector-based policies that negate the inclusiveness, interconnectedness, and interlinkages of the WEF resources. Current empirical models focus on individual goals, addressing them from a sector, conceptual, or normative perspective.^5^^,^^21^

An argument that has been suggested is the need to include the environment, health, sanitation, or ecosystems in the WEF nexus as they are regarded as integral components to human wellbeing.^45^^,^^87^^,^^88^ This has witnessed the emergence of WEF nexus derivatives like WEFE, WEFH, WEFEH and WEFS, which add the environment, health, and sanitation components to the WEF nexus, respectively. This school of thought tends to forget that water-energy and food (WEF) resources are the major resources that are both the culprits and victims of the grand challenges faced by humankind today.^56^ If humankind takes care of the three resources and improves their management, the other systems are also taken of.^77^^,^^79^ Changes and developments being made to the three resources are the major causes of climate change.^56^ The negative changes occurring to the environment, health and sanitation and the related challenges are only trade-offs resulting from the sector-based and linear models being used today to manage the interconnected WEF resources.^89^ This is where the WEF nexus is critical as it timely identifies trade-offs and synergies for timely intervention.^21^ As an example, there is a call to increase the irrigated area for the agriculture sector to be able to meet the growing food demands of an increasing population.^24^^,^^90^ Although this is a noble call, increasing the irrigated area requires more energy to draw the water to the agricultural field,^91^ which may produce non-renewable energy which causes environmental risks and pollution (environmental trade-offs). Conversely, increasing the irrigated area increases water demand to irrigate the crops,^92^ yet water resources are already scarce (water-related trade-offs). This will also destroy the natural habitat to create more land for agriculture,^15^ resulting in wild animals living closer to human beings in search of food, risking the emergence of zoonotic diseases (health trade-offs). Irrigation expansion also increase the risk of water-borne diseases like malaria (health trade-offs).^93^ Therefore, WEF resources are key to sustainable development and the challenges manifesting from the other systems are simply trade-offs of what is happening within the WEF resources. Taking care of the three resources, takes care of the rest. Therefore, there no need to include the environmental and health components or any other components to the WEF nexus as they are already embedded into the WEF nexus and are addressed through the identification of the trade-offs and synergies.

One hindrance to the realisation of the SDGs has been the lack of an integrated and all-inclusive governance framework, which has resulted in a clear and apparent disconnection between global aspirations and reality, now that the SDGs have passed the halfway mark without meaningful progress.^62^^,^^94^ As already alluded to, the interconnectedness of the WEF resources and their multi-sectoral reference provides the key to sustainable development. However, there is a need for policy integration and alignment to drive the 2030 Agenda.^95^ There is, therefore, a huge gap in providing pathways to align sectoral policies and design a framework to guide the cross-sectoral coordination, development, and management of resources. Such a framework could provide an inclusive roadmap toward a more integrated and cross-sectoral strategy toward achieving the 2030 Global Agenda.

Failure to recognize the interconnectedness of the SDGs themselves, and the lack of an integrated governance framework pose the greatest risk to achieving the SDGs, besides the emergence of novel infectious diseases. There is a strong link between the SDGs and the WEF nexus through the related indicators. Therefore, when WEF resources are efficiently managed and equitably distributed, they play an important role in strengthening the resilience of socio-economic and environmental systems. Therefore, achieving the desired outcomes requires integrating all the goals into national development plans, supported by an inclusive governance framework that promotes an enabling environment for collective action, ensuring stakeholders are held accountable for addressing emerging and complex trade-offs between the goals. Focusing on a few goals is often associated with systemic risks resulting from strategies that lead to suboptimal efficiencies in one sector at the expense of other equally important sectors, derailing efforts on economic and sustainable development.^6^^,^^15^ These challenges are exacerbated by climate change, land degradation, growing global population, increasing urbanization, and shifting consumption patterns, intensifying global resource demand.^96^ Furthermore, the challenges are intensifying at a time of rapid global change, a term that refers to biophysical, ecosystem and socio-economic changes that are altering the functioning of Earth systems on a planetary scale.^97^

Shifting from the normal way of living entails transitioning from a linear system to a circular economy that allows resources to stay longer in use and reduces environmental waste. Some of the key actions needed to achieve the SDGs include (1) reducing the huge gap between science and policy, and policy and implementation of novel technologies and innovations, (2) innovative financing models to support research and implementation, (3) promoting partnerships and collaboration among stakeholders and break the silos, (4) promoting local solutions to global challenges, (5) leveraging digital and climate-smart technologies that enhance adaptation and resilience strategies, and (6) promoting capacity development Therefore, the following is recommended to guide policy and decision-makers in achieving sustainable development.

(1) The SDGs are a systemic framework that acknowledges that action in one sector without considering the broad interlinkages with the other closely interlinked sectors will affect outcomes in the other sectors. Therefore, science should provide WEF nexus practical empirical evidence linking the approach with SDGs. This should be accompanied by the development of nexus-based scenarios that enhance resilience-building initiatives across scales. The presence of such evidence and demonstration of what the nexus can do will catalyze the operationalization of the WEF nexus as a pathway to achieve developmental goals.
(2) There is also an urgent need to foster policy coherence among the WEF sectors to drive integrated SDGs and WEF sectors’ implementation and operationalization. The lack of a coherent and cross-sectoral policy framework has been the missing link between WEF nexus implementation and achieving the SDGs. Thus, achieving the SDGs requires integrated, transformative and cross-sectoral national, regional, and international governance structures that coherently contribute to the SDGs and underlying targets. Policy coherence is critical in achieving the SDGs and operationalizing the WEF nexus as it promotes policies in different sectors to speak to each other and achieve development goals.
(3) As the SDGs are about people and the planet and the WEF nexus is about holistic and integrated management of interlinked resources, it is paramount to consider socio-ecological and economic systems in any global decisions that impact the Earth. Linking the SDGs and the WEF nexus in development programmes provides pathways that prioritize people and the planet, considering inclusivity to all stakeholders without leaving anyone behind.
(4) The SDGs are an opportunity for comprehensive, transformative change at the global scale, a platform for negotiating and implementing accords that benefit humankind and the Earth. As the United Nations General Assembly (UNGA) puts it,” a shared vision for global development toward a sustainable economy, society, and environment”.^39^ Thus, adopting the WEF nexus and other transformative approaches like the circular economy, just transition, sustainable food systems and one health, among others can catalyze the cause of the SDGs.
(5) As transformative solutions are being offered to drive the SDGs, it is important to formulate policies around those transformative solutions at national, regional, and global scales. A concrete example is shifting from the norm and transitioning from the linear economic model to a more sustainable circular economy. These solutions can inspire the formulation of coherent, progressive policies that contribute to the realization of “peace and prosperity for all, now and into the future”.

## Conclusions

The SDGs were formulated to reduce poverty, inequality, and conflict and conserve ecosystem services critical to human wellbeing and livelihoods. However, by the halfway mark, more than half of the world population has been left behind as progress on more than 50% of the SDG targets has been classified as weak and insufficient, and 30% have stalled or gone into reverse mode, including key targets on poverty, hunger, and climate. The first half of the SDGs has shown that the global aspirations of a sustainable planet by 2030 may be a far cry if humankind continues with the business-*as*-usual approach amid the evidence of a gloomy and disastrous future. This study developed a multisectoral and multidisciplinary framework to guide policy and decision-makers when formulating coherent strategies to implement the SDGs. The study highlights the prospects of managing WEF resources sustainably under climate change, environmental degradation, and increasing demand. The other 14 Goals are all linked to the water, energy, and food goals. Humankind needs to quadruple its efforts if the aspirations are to be achieved by 2030, as most of the targets’ first half has shown retrogressions. Key to the realization of the SDGs is the recognition of the interconnectedness of the SDGs and their targets and indicators and the need for an inclusive, cross-sectoral, and integrated policy framework to guide SDGs implementation and WEF nexus operationalization, among other factors that include funding, advocacy and education. The SDGs are measured through interlinked targets and indicators; therefore, achieving them individually or from a sector perspective could result in undesired outcomes. Failure to acknowledge the interconnectedness of the SDGs and the lack of an integrated governance framework has posed the greatest risk to the realization of the SDGs, besides the emergence of novel infectious diseases. When WEF resources are efficiently managed and equitably distributed, they play an important role in strengthening the resilience of socio-economic and environmental systems. The proposed framework is the initial phase toward the simultaneous realization of the SDGs.

## Acknowledgments

This work forms part of the Sustainable and Health Food Systems (SHEFS) Program, supported through the Welcome Trust’s Our Planet, Our Health Programme (grant no 205200/Z/16/Z), and the Water Research Commission through the Research, Development, and Innovation Funding.

## Author contributions

Conceptualisation, L.N., and T.M.; investigation, L.N., S.M., S.L., and T.M.; resources, L.N., S.M., S.L., and T.M.; writing—original draft, L.N.; writing—review and editing L.N., S.M., S.L., and T.M.; funding acquisition, S.M., S.L., and T.M.; supervision S.L.

## Declaration of interests

The authors declare no competing interests.
